# Prediction of life-story narrative for end-of-life surrogate’s decision-making is inadequate: a Q-methodology study

**DOI:** 10.1186/s12910-019-0368-8

**Published:** 2019-05-03

**Authors:** Muhammad M. Hammami, Kafa Abuhdeeb, Muhammad B. Hammami, Sophia J. S. De Padua, Areej Al-Balkhi

**Affiliations:** 1 Clinical Studies and Empirical Ethics Department, King Faisal Specialist Hospital and Research Centre, P O Box # 3354 (MBC 03), Riyadh, 11211 Saudi Arabia; 2 Alfaisal University College of Medicine, Riyadh, Saudi Arabia; 3 Florida Atlantic University, Florida, USA

**Keywords:** End-of-life choices, Q-methodology, Surrogate decision-making, Substituted judgment, Life-story narrative, Surrogate decision-making confidence, Acceptability of healthcare- outcomes, Decision-control preference

## Abstract

**Background:**

Substituted judgment assumes adequate knowledge of patient’s mind-set. However, surrogates’ prediction of individual healthcare decisions is often inadequate and may be based on shared background rather than patient-specific knowledge. It is not known whether surrogate’s prediction of patient’s integrative life-story narrative is better.

**Methods:**

Respondents in 90 family pairs (30 husband-wife, 30 parent-child, 30 sibling-sibling) rank-ordered 47 end-of-life statements as life-story narrative measure (Q-sort) and completed instruments on decision-control preference and healthcare-outcomes acceptability as control measures, from respondent’s view (respondent-personal) and predicted pair’s view (respondent-surrogate). They also scored their confidence in surrogate’s decision-making (0 to 4 = maximum) and familiarity with pair’s healthcare-preferences (1 to 4 = maximum). Life-story narratives’ prediction was examined by calculating correlation of statements’ ranking scores between respondent-personal and respondent-surrogate Q-sorts (projection) and between respondent-surrogate and pair-personal Q-sorts before (simulation) and after controlling for correlation with respondent-personal scores (adjusted-simulation), and by comparing percentages of respondent-surrogate Q-sorts co-loading with pair-personal vs. respondent-personal Q-sorts. Accuracy in predicting decision-control preference and healthcare-outcomes acceptability was determined by percent concordance. Results were compared among subgroups defined by intra-pair relationship, surrogate’s decision-making confidence, and healthcare-preferences familiarity.

**Results:**

Mean (SD) age was 35.4 (10.3) years, 69% were females, and 73 and 80% reported ≥ very good health and life-quality, respectively. Mean surrogate’s decision-making confidence score was 3.35 (0.58) and 75% were ≥ familiar with pair’s healthcare-preferences. Mean (95% confidence interval) projection, simulation, and adjusted-simulation correlations were 0.68 (0.67–0.69), 0.42 (0.40–0.44), and 0.26 (0.24–0.28), respectively. Out of 180 respondent-surrogate Q-sorts, 24, 9, and 32% co-loaded with respondent-personal, pair-personal, or both Q-sorts, respectively. Accuracy in predicting decision-control preference and healthcare-outcomes acceptability was 47 and 52%, respectively. Surrogate’s decision-making confidence score correlated with adjusted-simulation’s correlation score (rho = 0.18, *p* = 0.01). There were significant differences among the husband-wife, parent-child, and sibling-sibling subgroups in percentage of respondent-surrogate Q-sorts co-loading with pair-personal Q-sorts (38, 32, 55%, respectively, *p* = 0.03) and percent agreement on healthcare-outcomes acceptability (55, 35, and 67%, respectively, *p* = 0.002).

**Conclusions:**

Despite high self-reported surrogate’s decision-making confidence and healthcare-preferences familiarity, family surrogates are variably inadequate in simulating life-story narratives. Simulation accuracy may not follow the next-of-kin concept and is 38% based on shared background.

**Electronic supplementary material:**

The online version of this article (10.1186/s12910-019-0368-8) contains supplementary material, which is available to authorized users.

## Background

Respect for patient’s autonomy and right of self-determination dominate modern healthcare decision-making. The concept of precedent autonomy [1] indicates that personal choices ought to inform healthcare decisions for decisionally-incompetent patients, through patient’s advance directives and, if not available, substituted judgment by patient-designated or next-of-kin surrogate. According to the substituted judgment standard, surrogate decisions should be based on what the patients would have wanted rather than what the surrogates prefer for themselves or for their loved ones, what they think is in the best interests of the patients, or what they think most people would want [2].

The ethical validity of substituted judgment has been debated based on philosophical considerations [1, 3] and empirical findings [4–8]. Two systematic reviews found low to moderate prediction accuracy [4, 5]. Further, it is not clear how much of the prediction is based on patient-specific knowledge as compared to shared background at the level of the family or society. However, most of the reviewed studies focused on life-saving interventions and binary-decision scenarios; while in real life, substituted judgment usually involves a variety of concurrent issues such as life quality, life quantity, connectedness, transcendence, information disclosure, decision-control, and coping [9]. Moreover, there is often more than one acceptable answer, continuum rather than binary choices, and balancing rather than prioritizing.

Several alternatives to substituted judgment as a basis of surrogate’s decision-making have been proposed [10], including a population-based treatment indicator [11], family autonomy, rooted in the Eastern principle of familism [12, 13], the Golden Rule -“Do to others what you want them to do to you.” [14], and patient’s integrative life-story narrative.

The integrative life-story approach is based on understanding the personhood of the patient through eliciting a narrative about the patient’s life experiences, beliefs, and values [15–20]. It balances rather than rigidly prioritizes and considers not only the patient’s prior wishes but also the patient’s life-long dispositions, relationships, decisions, decision-making processes, risk-taking attitude, preferences, interests, and expressed beliefs and values.

We have used a Q-methodology based instrument to capture an integrated life-story narrative in relation to end-of-life issues [9, 21, 22]. Q-methodology, a special type of by-person exploratory factor analysis, is a process whereby respondents model their point of view by rank-ordering opinion statements into piles (Q-sort) along a continuum defined by certain instructions [23]. Using the Q-sorts as variables, Q-methodology finds cross-cutting similarities and produces grouping of like-minded respondents who rank-order the statements into similar arrangements [24].

The main aims of this study were to evaluate surrogates’ prediction of family members’ life-story narrative in relation to end-of-life issues and the extent it is based on family member-specific knowledge rather than shared background. Since prediction accuracy may depend on type of relationship, it was specifically examined in three categories of family pairs, husband-wife, parent-child, and sibling-sibling. We determined surrogate’s accuracy in predicting decision-control preference and healthcare-outcomes acceptability as control measures. We also explored whether prediction accuracy varies according to intra-pair relationship, intra-pair familiarity, surrogate’s decision-making confidence, or demographic factors.

## Methods

The study was conducted between April 2016 and May 2017 according to the Declaration of Helsinki. It was approved by the Research Ethics Committee (REC) of the King Faisal Specialist Hospital and Research Center (KFSH&RC). All respondents provided verbal informed consent as approved by the REC.

### Volunteer sample

KFSH&RC employees and KFSH&RC outpatients and their companions were invited to participate. Eligibility criteria were: having more than high school education, being more than 18 years old, and being able to understand study purpose and perform its procedures. The sample size of 30 husband-wife pairs, 30 parent-child pairs, and 30 sibling-sibling pairs (total 90 pairs or 180 respondents) was based on convenience, limitation of the Q-methodology program (up to 125 Q-sorts), and theoretical association between type of relationship and prediction accuracy.

Respondents in each family pair separately sorted the Q-set (see below) and completed instruments (pen and paper survey) on decision-control preference and compromised healthcare-outcomes acceptability according to their personal view (respondent-personal) and predicted view of their participating family member (respondent-surrogate). They also scored their surrogate’s decision-making confidence and familiarity of healthcare-preferences of their participating family member and provided demographic data.

### Q-set sorting

The development and validation of the Q-set were reported previously [9]. Respondents arranged the Q-set statements into graded priority and dis-priority, using a sorting sheet that has 9 categories (1 = extreme dis-priority, 9 = extreme priority) with symmetrically distributed slots. The Q-set (Additional file 1) has 47 statements distributed in 8 end-of-life thematic domains (each with 5 to 8 statements). Time spent to complete each Q-sort was recorded. Completeness of Q-sorting was checked immediately and respondents were asked to correct any identified mistakes without any further input from the study coordinator.

### Decision-control preference instrument

Decision-control preference was assessed using the patient–family decision making version of the decision control preferences scale [25], which consists of captioned illustrations depicting five decision-control options (from independent, through shared, to reliant decision-making) under conscious and nonconscious scenarios. Respondents were asked to imagine that they have (or that their participating family member has) a large thyroid nodule that could be benign or cancerous and that the only way to know is to have thyroid surgery, which may have certain complications. Using captioned illustrations, they were asked to indicate their preference (or predicted preference of their participating family member) for family involvement in decision-making.

### Compromised healthcare-outcomes acceptability instrument

Acceptability of compromised healthcare-outcomes was assessed using the values of life-sustaining treatment outcomes instrument [6], which assesses individual’s threshold for unacceptable outcomes of life-sustaining treatment. Respondents were asked to imagine that they became seriously ill at the hospital and to think about conditions that for them would be worse than death and thus they would want their family member to make a decision to stop life-sustaining treatment and focus on treatment to make them as comfortable as possible. Respondents were presented with four outcomes (“cannot recognize family or friends”, “only respond to pain and yet in untreatable pain most of the time”, “can no longer control bowels”, and “have to live in a nursing home until death”) and asked whether the outcome would be or is predicted to be, “acceptable”, “unacceptable”, or “unsure.” to them or to their participating family member, respectively.

### Surrogate’s decision-making confidence scale

Confidence in surrogate decision-making was assessed using a previously published scale [6]. The scale consists of five items with response options from 0 (not confident at all) to 4 (very confident). The 5 items are: “I understand what my family member’s preferences are.”, “I can make a decision for my family member as to what treatment he/she should have, even in a highly stressful situation”, “I can ask questions to get the facts about benefits or risks of each medical choice without feeling discouraged.”, “I can handle unwanted pressure from others, such as other family members or healthcare providers, in making decisions for my family member.” and “I can communicate with doctors and nurses about my family member’s wishes.” Scale score was calculated as the mean score of the five items.

We also collected data on the following: age, sex, living arrangement, employment, religiosity (1 = most religious to 5 = least religious), general health now (excellent, very good, good, fair, poor) and compared to one year ago (much better now, somewhat better now, about the same, much worse now), ability to carry out essential daily activities (without help, with some help, completely dependent), pain in last month (none, a little pit, moderate, quite a pit, extreme), life quality (excellent, very good, good, fair, bad), response to a satisfaction statement, “If I could live my life over, I would change nothing” (strongly agree, agree, neutral, disagree, strongly disagree), death experience (last year, last 5 years, none in last 5 years), intra-pair relationship type, and degree of familiarity with participating family member healthcare-preferences (very familiar, familiar, somewhat familiar, not familiar at all).

The study was conducted in one session in three steps and study forms were collected after each step. Step one: Q-sorting, acceptability of healthcare outcomes instrument, and decision-control preference instrument for self. Step two: demographic and health status data, surrogate’s decision-making confidence scale, familiarity with participating family member healthcare-preferences, and expected familiarity of participating family member with respondent’s healthcare-preferences. Step three: Q-sorting, acceptability of healthcare-outcomes instrument, and decision-control preference instrument for participating family member. Respondents were reimbursed for time and inconvenience.

### Analysis

Correlations of ranking scores of individual statements between respondent-personal and respondent-surrogate Q-sorts (projection) and between respondent-surrogate and pair-personal Q-sorts before (simulation) and after controlling for correlation with respondent-personal scores (adjusted-simulation) were determined using Pearson’s correlation coefficient. We used z-transformation to estimate mean correlation coefficient [26]. We compared mean correlation coefficients by first calculating the combined standard error (square root of the sum of squares of the separate standard errors) and then performing the z test and determining the 95% confidence interval (CI) [27].

We used PCQ for Windows (PCQ Software, Portland, OR, USA) to analyze the Q-sorts. Extracted centroids were Varimax (variance maximizing) rotated to find a solution for which each Q-sort (respondent) has only a small number of large loadings. Varimax rather than manual rotation was used to avoid bias. Factor loading was determined to establish the extent to which each Q-sort is correlated with each of the identified factors. Loading in excess of 0.38 (*p* < 0.01) was considered significant. The percentages of respondent-surrogate Q-sorts that significantly co-loaded with respondent-personal Q-sorts, with pair-personal Q-sorts, with both, or with neither were determined for 7-factor solution (as well as for 5-factor and 9-factor solutions in terms of sensitivity analysis) and compared using chi-squared test.

Prediction accuracy of life-story narrative was examined by calculating correlation of Q-set individual statements’ ranking scores between respondent-personal and respondent-surrogate Q-sorts (projection) and between respondent-surrogate and pair-personal Q-sorts before (simulation) and after controlling for correlation with respondent-personal scores (adjusted-simulation). Prediction accuracy was also examined by comparing the percentage of respondent-surrogate Q-sorts that co-loaded with pair-personal Q-sorts to the percentage of respondent-surrogate Q-sorts that co-loaded with respondent-personal Q-sorts. Mean scores of the surrogate’s decision-making confidence scale were calculated per item and per scale and compared among the three subgroups and according to sex and religion using analysis of variance (ANOVA)/independent t-test. Familiarity with healthcare-preferences, decision-control preferences, and percentages of respondent-surrogate Q-sorts co-loading with pair-personal Q-sorts were compared among the three subgroups using chi-squared test. Prediction accuracy of decision-control preference and healthcare-outcomes acceptability was calculated as percent concordance and compared among the three subgroups and according to surrogate’s decision-making confidence score (>3.5 vs. ≤3.5), healthcare-preferences familiarity (familiar/very familiar vs. somewhat familiar/not at all familiar), sex, and religion using chi-squared test. Mean surrogate’s decision-making confidence scores were compared according to accuracy in predicting decision-control preference, accuracy in predicting healthcare-outcomes acceptability, and co-loading of respondent-surrogate Q-sorts with pair-personal Q-sorts using independent t-test. Associations of surrogate’s decision-making confidence score, healthcare-preferences familiarity score, adjusted-simulation correlation score, and age was examined using Spearman correlation. Statistical analysis was performed (by MMH) using IBM SPSS Statistics version 21 software, respectively. 2-tail unadjusted *p* values are reported.

## Results

One hundred eighty adults in three equal subgroups (husband-wife, parent-child, or sibling-sibling) participated in the study. Respondents’ demographics, religiosity, health status, life quality, life satisfaction, and death experience in family/close friends are summarized in Table 1.

**Table 1:** Characteristics of study respondents

	Total	Husband-wife	Parent-child	Sibling-sibling
Age-mean (SD), yr.	35.4(10.3)	35.8 (12.0)	38.8 (14.5)	31.6 (6.9)
Sex-no. (%)				
Males	55 (31)	30 (50)	8 (13)	17 (28)
Females	125 (69)	30 (50)	52 (87)	43 (72)
Living arrangement-no. (%)				
With spouse	104 (58)	60 (100)	29 (48)	17 (28)
With parents	51 (28)	–	26 (43)	24 (40)
With children only	6 (3)	–	4 (7)	1 (2)
With siblings only	13 (7)	–	–	13 (22)
Alone	5 (3)	–	1 (2)	4 (7)
Others	1 (1)	–	–	1 (2)
Employment-no. (%)				
Student	17 (9)	–	13 (22)	4 (7)
Employed	148 (82)	57 (95)	38 (63)	53 (88)
Self employed	1 (1)	–	–	1 (2)
Not employed	5 (3)	–	3 (5)	2 (3)
House wife	9 (5)	3 (5)	6 (10)	–
Religiosity- no. (%)				
1 (most)	36 (20)	16 (27)	14 (23)	6 (10)
2	51 (28)	19 (32)	14 (23)	18 (30)
3	71 (39)	17 (28)	22 (37)	32 (53)
4	17 (9)	6 (10)	8 (13)	3 (5)
5 (least)	5 (3)	2 (3)	2 (3)	1 (2)
Religion- no. (%)				
Christian	95 (53)	39 (65)	28 (47)	28 (47)
Muslim	85 (47)	21 (35)	32 (53)	32 (53)
Health now- no. (%)				
Excellent	45 (25)	11 (18)	19 (32)	15 (25)
Very good	87 (48)	34 (57)	24 (40)	29 (48)
Good	44 (24)	15 (25)	14 (23)	15 (25)
Fair	2 (1)	–	1 (2)	1 (2)
Poor	2 (1)	–	2 (3)	–
Health compared to one year ago- no. (%)				
Much better now	53 (29)	19 (32)	14 (23)	20 (33)
Somewhat better now	30 (17)	12 (20)	9 (15)	9 (15)
About the same	83 (46)	26 (43)	33 (55)	24 (40)
Somewhat worse now	14 (8)	3 (5)	4 (7)	7 (12)
Much worse now	–	–	–	–
Essential daily activities-no. (%)				
Without help	162 (91)	52 (87)	56 (95)	54 (90)
With some help	2 (1)	–	1 (2)	1 (2)
Completely dependent	15 (8)	8 (13)	2 (3)	5 (8)
Pain in last month-no. (%)				
None	82 (46)	28 (47)	25 (42)	29 (48)
A little bit	53 (29)	18 (30)	16 (27)	19 (32)
Moderate	32 (18)	10 (17)	14 (23)	8 (13)
Quite a bit	12 (7)	4 (7)	4 (7)	4 (12)
Extreme	1 (1)	–	1 (2)	–
Life quality- no. (%)				
Excellent	55 (31)	22 (37)	18 (30)	15 (25)
Very good	88 (49)	29 (48)	29 (48)	30 (50)
Good	35 (19)	8 (13)	13 (22)	14 (23)
Fair	2 (1)	1 (2)	–	1 (2)
Bad	–	–	–	–
Response to “If I could live my life over, I would change nothing.”- no. (%)				
Strongly agree	22 (12)	6 (10)	9 (15)	7 (12)
Agree	62 (34)	21 (35)	20 (33)	21 (35)
Neutral	35 (19)	13 (22)	13 (22)	9 (15)
Disagree	49 (27)	18 (30)	12 (20)	19 (32)
Strongly disagree	12 (7)	2 (3)	6 (10)	4 (7)
Death experience in family/close friends-no. (%)				
Last year	57 (32)	15 (25)	13 (22)	29 (48)
Last 5 years	67 (37)	22 (37)	28 (47)	17 (28)
None in last 5 years	56 (31)	23 (38)	19 (32)	14 (23)

Percentages may not add to 100% due to rounding

### Confidence in surrogate’s decision-making and familiarity with healthcare-preferences

Confidence scores in surrogate’s decision-making in the three subgroups are presented in Table 2 per scale item and per scale. On a 5-point scale (zero = not confident at all to 4 = very confident), mean (SD) scale score for all respondents was 3.35 (0.58). There was significant difference among the three subgroups (*p* = 0.008), mean scale score was higher in the husband-wife subgroup (3.54 (0.43) compared to the parent-child subgroup (mean difference (95% CI) 0.23 (− 0.01–0.48), *p* = 0.07) and to the sibling-sibling subgroup (mean difference 0.32 (0.07–0.57), *p* = 0.007). Scores were also higher in the husband-wife subgroup for each of the 5 items.

**Table 2:** Surrogate’s decision-making confidence and healthcare-preferences familiarity

	Total	Husband-wife	Parent-child	Sibling-sibling
Surrogate’s decision-making confidence score-mean (SD) ^a^				
Item 1: I understand what my family member’s preferences are	3.34 (0.74)	3.53 (0.57)	3.22 (0.89)	3.28 (0.69)
Item 2: I can make a decision for my family member as to what treatment he/she should have, even in a highly stressful situation	3.22 (0.86)	3.47 (0.68)	3.12 (0.92)	3.07 (0.92)
Item 3: I can ask questions to get the facts about benefits or risks of each medical choice without feeling discouraged	3.40 (0.82)	3.57 (0.59)	3.48 (0.79)	3.15 (0.99)
Item 4: I can handle unwanted pressure from others, such as other family members or healthcare providers, in making decisions for my family member	3.24 (0.81)	3.42 (0.56)	3.18 (0.81)	3.12 (0.98)
Item 5: I can communicate with doctors and nurses about my family member’s wishes	3.56 (0.78)	3.70 (0.56)	3.52 (0.93)	3.47 (0.79)
Decision-making confidence scale ^b^	3.35 (0.58)	3.54 (0.43)	3.30 (0.61)	3.22 (0.65)
Familiarity of respondents with participating family member’s healthcare-preferences-no. (%)				
Very familiar	77 (43)	28 (47)	31 (52)	18 (30)
Familiar	58 (32)	18 (30)	15 (25)	25 (42)
Somewhat familiar	42 (23)	14 (23)	14 (23)	14 (23)
Not familiar at all	3 (2)	–	–	3 (5)
Predicted familiarity of participating family members with respondents’ healthcare-preferences-no. (%)				
Very familiar	73 (41)	24 (40)	33 (55)	16 (27)
Familiar	63 (35)	21 (35)	15 (25)	27 (45)
Somewhat familiar	42 (23)	15 (25)	11 (18)	16 (27)
Not familiar at all	2 (1)	–	1 (2)	1 (2)

^a^ Respondents scored their confidence in each of the 5 items in relation to their participating family member on a 5-point scale (zero = not confident at all to 4 = very confident). ^b^ P value comparing the three subgroups is 0.008. Percentages may not add to 100% due to rounding

Seventy five percent of the 180 respondents reported that they are familiar/very familiar with their participating family member’s healthcare-preferences and 76% reported that their participating family members are familiar/very familiar with their healthcare-preferences (Table 2). There were no significant differences among the three subgroups.

Familiarity with healthcare-preferences was coded on a 4-point scale (1 = very familiar to 4 = not familiar at all). There was significant correlation between surrogate’s decision-making confidence score and healthcare-preferences familiarity score (rho − 0.336, *p* < 0.001).

### Prediction accuracy of life-story narrative

**Fig. 1:**
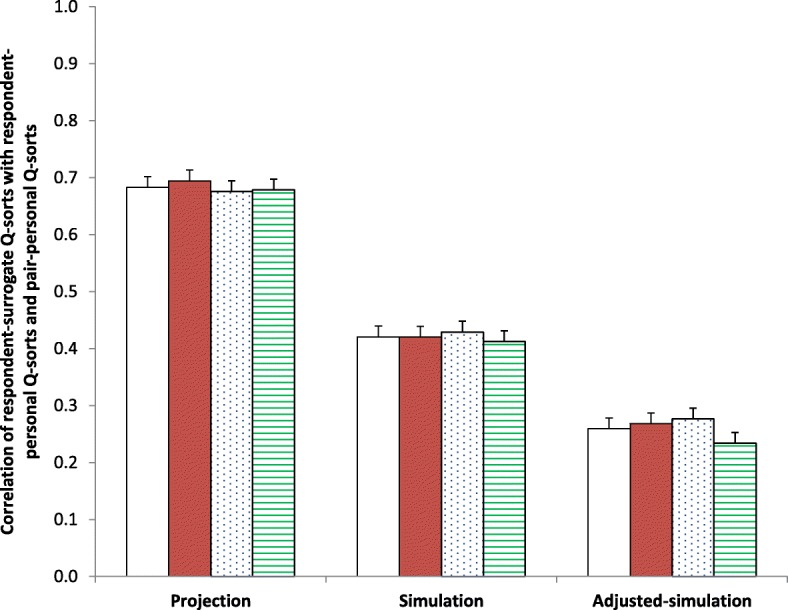
Correlation of respondent-surrogate Q-sorts with respondent-personal Q-sorts and pair-personal Q-sorts. Bars represent mean (SE) correlation of statements’ ranking scores between respondent-personal and respondent-surrogate Q-sorts (projection) and between respondent-surrogate and pair-personal Q-sorts before (simulation) and after controlling for correlation with respondent-personal scores (adjusted-simulation). Open bars, all 90 family pairs. Solid red bars, 30 husband-wife pairs. Blue dotted bars, 30 parent-child pairs. Green stripes bars, 30 sibling- sibling pairs

Overall, mean (95% CI) projection, simulation, and adjusted-simulation correlation scores were 0.68 (0.67–0.69), 0.42 (0.40–0.44), and 0.26 (0.24–0.28), respectively. The difference between projection and simulation correlation scores was significant (0.26 (0.23–0.30), p < 0.001). As shown in Fig. 1, projection, simulation, and adjusted-simulation correlation scores were similar among the three subgroups. However, adjusted-simulation was better by children compared to parents (correlation score 0.48 vs. 0.37, *p* = 0.006) and by wives compared to husbands (correlation score 0.46 vs. 0.38, *p* = 0.07).

Q-methodology produces grouping of like-minded respondents whose Q-sorts co-load on the same factor. We determined within-pair concordance, i.e., co-loading of respondent-surrogate Q-sorts and pair-personal Q-sorts on the same factor. As a control, we determined co-loading of respondent-surrogate Q-sorts with respondent-personal Q-sorts. The Q-methodology was applied to the three subgroups separately (Table 3). Using 7-factor extraction solution, the seven factors accounted for 48 to 53% of the total variance in the three subgroups (Eigen values 56.5 to 63.0). Out of the 180 respondent-surrogate Q-sorts, about 24% co-loaded with respondent-personal Q-sorts only, 9% co-loaded with pair-personal Q-sorts only, 32% co-loaded with both, and 34% did no co-load with either. It was 1.4 to 2.7 times more likely for respondent-surrogate Q-sorts to co-load with respondent-personal Q-sorts than with pair-personal Q-sorts, depending on whether co-loading with both was included. Within-pair concordance was significantly different among the three subgroups (*p* = 0.03). It was about 55% in the sibling-sibling subgroup compared to 32% in the parent-child subgroup (*p* = 0.01) and to 38% in the husband-wife subgroup (*p* = 0.07). As shown in Table 3, using 5-factor and 9-factor (instead of 7-factor) solutions gave similar overall results.

**Table 3:** Factor analysis of 180 surrogate and 180 personal Q-sorts

	Variance explained (%) / total Eigen value	Respondent-surrogate Q-sorts co-loading with:^a^			
		Respondent- personal Q-sorts only	Pair- personal Q-sorts only	Both	Neither
9-Factor solution					
Husband-wife	58/68.9	19 (32)	3 (5)	15 (25)	23 (38)
Parent-child	52/63.5	16 (27)	5 (8)	21 (35)	18 (30)
Sibling-sibling	58/67.6	12 (20)	5 (8)	22 (37)	21 (35)
Total		47 (26)	13 (7)	58 (32)	62 (34)
7-Factor solution					
Husband-wife	53/63.0	16 (27)	4 (7)	19 (32)	21 (35)
Parent-child	48/56.5	20 (33)	5 (8)	14 (23)	21 (35)
Sibling-sibling	52/61.6	8 (13)	8 (13)	25 (42)	19 (32)
Total		44 (24)	17 (9)	58 (32)	61 (34)
5-Factor solution					
Husband-wife	46/55.4	12 (20)	4 (7)	23 (38)	21 (35)
Parent-child	39/48.4	14 (23)	3 (5)	19 (32)	24 (40)
Sibling-sibling	46/54.7	9 (15)	3 (5)	26 (43)	22 (37)
Total		35 (19)	10 (6)	68 (38)	67 (37)

^a^Data represent number (%) of respondent-surrogate Q-sorts that co-loaded with respondent-personal Q-sorts only, pair-personal Q-sorts only, both, or neither. Percentages may not add to 100% due to rounding

### Prediction accuracy of individual statement’s ranking score

**Fig. 2:**
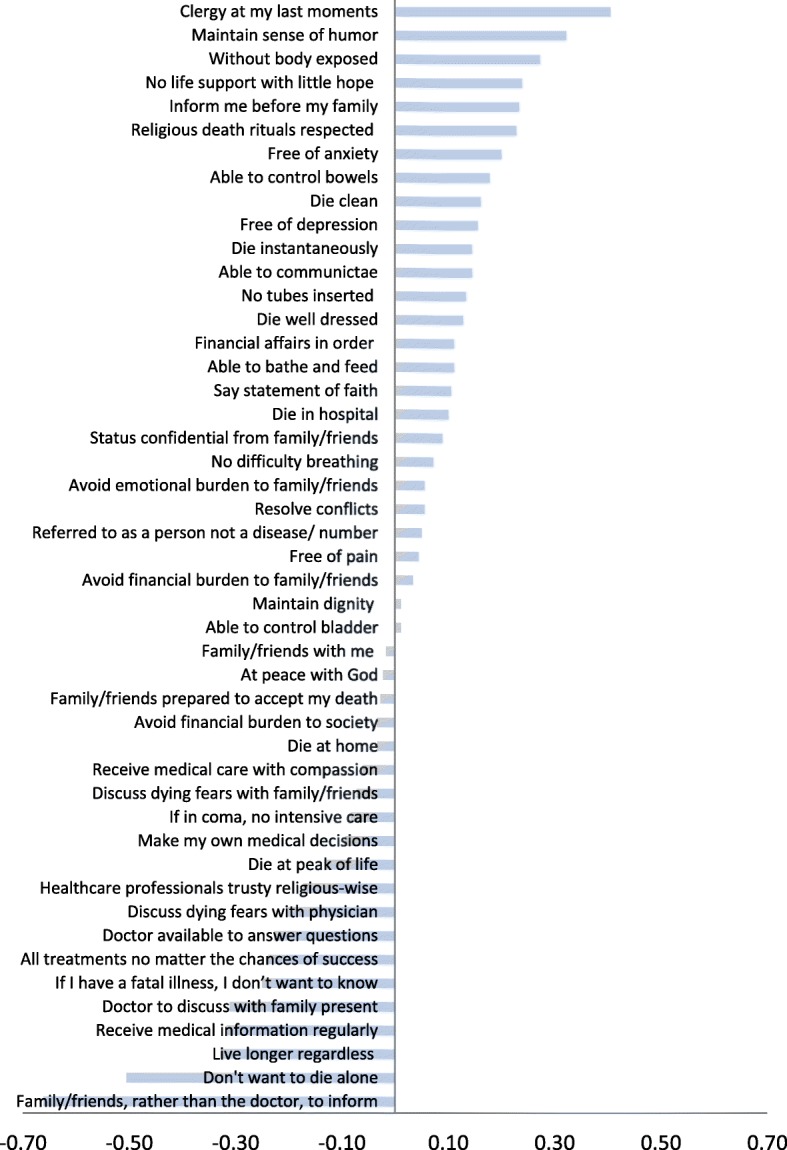
Intra-pair differences in forced-ranking scores of 47 end-of-life opinion statements. Bars represent mean of 180 intra-pair differences (personal minus surrogate) in ranking scores (on a scale of 1 to 9). Full description of the statements is presented in Additional file 1

In order to explore reasons for intra-pair discordance in Q-sorting, we examined prediction accuracy in rank-scoring of individual Q-sort statements. Overall, mean personal-surrogate difference in ranking scores (on a scale of 1 to 9) of the 47 statements ranged from − 0.66 to 0.41, consistent with the notion that averaging analysis may obscure important differences (Fig. 2). The most discordant statements were “I want my family/ friends rather than my doctor to inform me about my impending death”, “I don’t want to die alone.”, “I want to live longer regardless of my medical condition.”, “I want to receive medical information regularly from medical staff.”, and “I want my doctor to discuss any concerns relating to my illness and care in the presence of my family/friends.”, which received lower personal mean scores; and “I want to have a clergy with me at my last moments.”, “I want to die maintaining my sense of humor.”, and “I want to die without having my body exposed.”, which received higher personal mean scores.

**Fig. 3:**
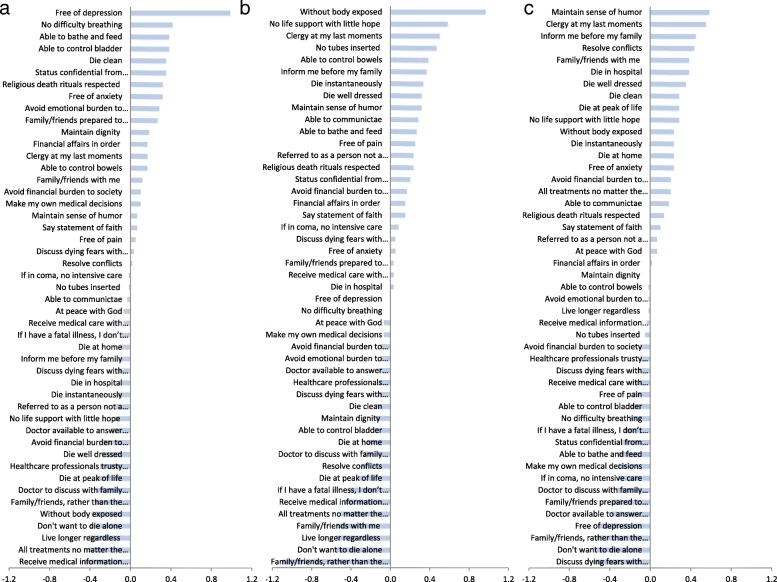
Intra-pair differences in forced-ranking scores of 47 end-of-life opinion statements per subgroup. Bars represent mean of 60 intra-pair differences (personal minus surrogate) in ranking scores (on a scale of 1 to 9). **a**, husband-wife subgroup. **b**, parent-child subgroup. **c**, sibling-sibling subgroup. Full description of the statements is presented in Additional file 1

When subgroups were analyzed separately, mean personal-surrogate difference in ranking scores ranged from − 0.45 to 0.98, from − 1.10 to 0.97, and from − 0.55 to 0.58, for the husband-wife, parent-child, and sibling-sibling subgroups, respectively, indicating a relatively wider range of discordance in the parent-child subgroup and a narrower range of discordance in the sibling-sibling subgroup. In the husband-wife subgroup the most discordant statement was “I want to die free of depression.”, which received higher personal mean score (Fig. 3a). This was also true when husbands and wives as surrogates were analyzed separately (Additional file 2).

In the parent-child subgroup, the most discordant statements were “I want my family/ friends rather than my doctor to inform me about my impending death.”, which received lower personal mean score and “I want to die without having my body exposed.”, which received higher personal mean score (Fig. 3b). However, when analyzed separately, this was true only when parents were predicting. When children were predicting, the most discordant statements were “I don’t want to die alone.”, which received lower personal mean score and “I want to have no tubes inserted into my body.”, which received higher personal mean score (Additional file 3).

Finally, in the sibling-sibling subgroup, the most disagreeable statements were “I want to die maintaining my sense of humor.” and “I want to have a clergy with me at my last moments.”, which received higher personal than surrogate mean scores; and “I want to discuss my fears about dying with my physician.” and “I do not want to die alone.”, which received lower personal mean scores (Fig. 3c).

### Prediction accuracy of decision-control preference

As a control measure, we examined surrogate’s accuracy in predicting decision-control preference. Respondents were asked to select one of five decision-control options, according to their own preference and according to the predicted preference of their participating family member, both under conscious and unconscious scenarios. The results are presented in Table 4.

**Table 4:** Decision-control preference under conscious and unconscious scenarios

	Total		Husband-wife		Parent-child		Sibling-sibling	
	Personal	Predicted	Personal	Predicted	Personal	Predicted	Personal	Predicted
Conscious scenario								
-I prefer to make the decisions about which tests or treatments I receive	41 (23)	37 (21)	12 (20)	9 (15)	11 (18)	11 (18)	18 (30)	17 (28)
-I prefer to make the final decision about which tests or treatments I receive after seriously considering my loved one’s opinions	49 (27)	40 (22)	12 (20)	8 (13)	18 (30)	16 (27)	19 (32)	16 (27)
-I prefer that my loved one and I share responsibility for deciding which tests or treatments I receive	78 (43)	84 (47)	32 (53)	35 (58)	27 (45)	26 (43)	19 (32)	23 (38)
-I prefer that my loved ones make the final decision about which tests or treatments I receive after seriously considering my opinion	7 (4)	14 (8)	3 (5)	7 (12)	1 (2)	4 (7)	3 (5)	3 (5)
-I prefer to leave all decisions about which tests or treatments I receive to my loved ones	5 (3)	5 (3)	1 (2)	1 (2)	3 (5)	3 (5)	1 (2)	1 (2)
Unconscious scenario								
-I prefer that my loved ones tell my doctor which tests or treatments to order for me based on my own personal wishes	24 (13)	26 (14)	9 (15)	9 (15)	7 (12)	6 (10)	8 (13)	11 (18)
-I prefer that my loved ones tell my doctor which tests or treatments to order for me based on my own personal wishes, after having seriously considered what they think is best	44 (24)	46 (26)	7 (12)	9 (15)	18 (30)	16 (27)	19 (32)	21 (35)
-I prefer that my loved ones tell my doctor which tests or treatments to order by equally weighing both my personal wishes and what my loved ones think is best	78 (43)	71 (39)	35 (58)	31(52)	18 (30)	21 (35)	25 (42)	19 (32)
-I prefer that my loved ones tell my doctor which tests or treatments to order for me based on what they think is best, after having seriously considered my own personal wishes	19 (11)	21 (12)	5 (8)	7 (12)	8 (13)	9 (15)	6 (10)	5 (8)
-I prefer that my loved ones tell my doctor which tests or treatments to order for me based on what my loved ones think is best	15 (8)	16 (9)	4 (7)	4 (7)	9 (15)	8 (13)	2 (3)	4 (7)

Data are number (%) of respondents who selected the option for themselves (personal) or predicted that their participating family member would have selected (predicted). Percentages may not add to 100% due to rounding

The most preferred option was shared decision-making (between family and patient or family and patient’s previously declared wishes), which was selected by 43% under each scenario. Preference for this option was highest in the husband-wife subgroup; 53% vs. 45% in the parent-child subgroup (*p* = 0.36) and 32% in the sibling-sibling subgroup (*p* = 0.02) under the conscious scenario, and 58% vs. 30% in the parent-child subgroup (*p* = 0.002) and 42% in the sibling-sibling subgroup (*p* = 0.07) under the unconscious scenario. Shared decision-making was also predicted to be the most preferred option by participating family members (47 and 39% under the conscious and unconscious scenarios, respectively).

**Fig. 4:**
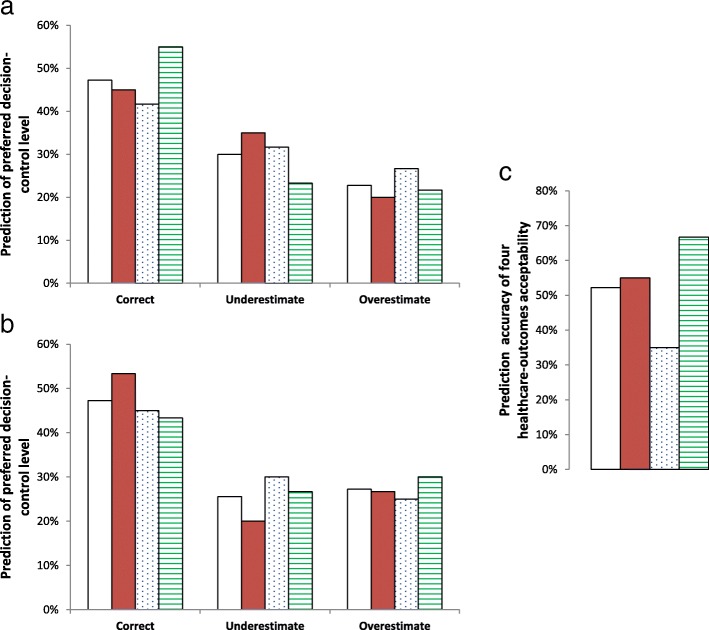
Prediction of preferred level of decision-control and of acceptability of compromised healthcare-outcomes. Bars represent mean percentages. **a**, prediction of preferred level of decision-control under the conscious scenario. **b**, prediction of preferred level of decision-control under the unconscious scenario. **c**, prediction accuracy of healthcare-outcomes acceptability. Open bars, all 90 family pairs. Solid red bars, 30 husband-wife pairs. Blue dotted bars, 30 parent-child pairs. Green stripes bars, 30 sibling-sibling pairs

As shown in Fig. 4a & b, overall prediction accuracy of decision-control preference was about 47% under both scenarios. Thirty percent and 26% underestimated and 23 and 27% overestimated the preferred level of decision-control under the conscious and unconscious scenarios, respectively. Although the sibling-sibling subgroup and the husband-wife subgroup were the most accurate under the conscious and unconscious scenario, respectively, the differences were not significant (*p* ≥ 0.14).

### Prediction accuracy of healthcare-outcomes acceptability

As a second control measure, we examined prediction accuracy of healthcare-outcomes acceptability. Respondents were asked to choose “acceptable”, “unacceptable”, or “unsure” in relation to four compromised healthcare-outcomes that for them (or as predicted for their participating family member) would be worse than death and under which they would want their family to make a decision to stop life-sustaining treatments and focus on comforting treatments. The results are summarized in Table 5.

**Table 5:** Acceptability of compromised healthcare-outcomes

	Total	Husband-wife	Parent-child	Sibling-sibling
Cannot recognize family or friends-no. (%)				
-Acceptable for self	28 (16)	8 (13)	15 (25)	5 (8)
Predicted acceptable for family member	25 (14)	9 (15)	11 (18)	5 (8)
-Unacceptable for self	142 (79)	52 (87)	39 (65)	51 (85)
Predicted unacceptable for family member	143 (79)	48 (80)	43 (72)	52 (87)
-Not sure for self	10 (6)	–	6 (10)	4 (7)
Not sure for family member	12 (7)	3 (5)	6 (10)	3 (5)
Only respond to pain and yet in untreatable pain most of the time-no. (%)				
-Acceptable for self	24 (13)	6 (7)	15 (25)	3 (5)
Predicted acceptable for family member	21 (12)	7 (12)	12 (20)	2 (3)
-Unacceptable for self	137 (76)	51 (85)	35 (58)	51 (85)
Predicted unacceptable for family member	144 (80)	48 (80)	42 (70)	54 (90)
-Not sure for self	19 (10.6)	3 (5)	10 (17)	6 (10)
Not sure for family member	15 (8.3)	5 (8)	6 (10)	4 (7)
Can no longer control bowels-no. (%)				
-Acceptable for self	20 (11.1)	4 (6.7)	13 (22)	3 (5)
Predicted acceptable for family member	18 (10.0)	4 (6.7)	11 (18)	3 (5)
-Unacceptable for self	146 (81)	51 (85)	40 (67)	55 (92)
Predicted unacceptable for family member	142 (79)	51 (85)	38 (63)	53 (88)
-Not sure for self	14 (8)	5 (8)	7 (12)	2 (3)
Not sure for family member	20 (11)	5 (8)	11 (18)	4 (7)
Have to live in a nursing home until death-no. (%)				
-Acceptable for self	21(12)	5 (8)	12 (20)	4 (7)
Predicted acceptable for family member	16 (9)	4 (7)	9 (15)	3 (5)
-Unacceptable for self	142 (79)	51 (85)	42 (70)	49 (82)
Predicted unacceptable for family member	149 (83)	50 (83)	46 (77)	53 (88)
-Not sure for self	17 (9)	4 (7)	6 (10)	7 (12)
Not sure for family member	15 (8.)	6 (10)	5 (8)	4 (7)

Percentages may not add to 100% due to rounding

Being unable to recognize family or friends was unacceptable to 79% of the 180 respondents, with smaller percentage in the parent-child subgroup (65% vs. 87% in the husband-wife subgroup (*p* = 0.006) and 85% in the sibling-sibling subgroup (*p* = 0.01)). Only being able to respond to pain and yet in untreatable pain most of the time was unacceptable to 76%, with smaller percentage in the parent-child subgroup (58% vs. 85% in the husband-wife and sibling-sibling subgroups (*p* = 0.001)). Being unable to control bowels was unacceptable to 81%, with smaller percentage in the parent-child subgroup (67% vs. 85% in the husband-wife subgroup (*p* = 0.02) and 92% in the sibling-sibling subgroup (*p* = 0.007)). Finally, having to live in a nursing home until death was unacceptable to 79%, with smaller percentage in the parent-child subgroup (70% vs. 85% in the husband-wife subgroup (*p* = 0.05) and 82% in the sibling-sibling subgroup (*p* = 0.14)).

As shown in Fig. 4c, prediction accuracy for the four outcomes combined was 52%, with significant difference among the three subgroups (*p* = 0.002). It was least in the parent-child subgroup (35% vs. 55% in the husband-wife subgroup (*p* = 0.03) and 67% in the sibling-sibling subgroup (*p* < 0.001). This was also true when prediction accuracy was separately evaluated for the individual outcomes. It was 72% in the parent-child subgroup (vs. 82% in the husband-wife and sibling-sibling subgroups, *p* = 0.20) for the “Can’t recognize family or friends.” outcome; 65% (vs. 80% in the husband-wife subgroup, *p* = 0.07 and 77% in the sibling-sibling subgroup, *p* = 0.2) for the “Only respond to pain and yet in untreatable pain most of the time.” outcome; 62% (vs 80.0% in the husband-wife and sibling-sibling subgroups, *p* = 0.03) for the “Can no longer control bowels.” outcome; and 70% (vs. 77% in the husband-wife subgroup, *p* = 0.4 and 90.0% in the sibling-sibling subgroup, *p* = 0.006) for the “Have to live in nursing home until death.” outcome.

### Association of prediction accuracy with surrogate’s decision-making confidence, healthcare-preferences familiarity, and demographic variables

There was significant correlation between surrogate’s decision-making confidence score and adjusted-simulation correlation score (rho 0.18, *p* = 0.01). However, there were no significant differences in mean surrogate’s decision-making confidence score between respondents whose respondent-surrogate and pair-personal Q-sorts co-loaded or did not co-load, between respondents who accurately predicted decision-control preference and respondents who did not, or between respondents who accurately predicted acceptability of all 4 healthcare-outcomes and respondents who did not (*p* = 0.41 to 0.89).

Respondents were divided into two groups according to their surrogate’s decision-making confidence score (84 with score >3.5 vs. 96 with score ≤ 3.5) or healthcare-preferences familiarity (135 familiar/very familiar vs. 45 somewhat familiar/ not at all familiar). There were no significant differences in terms of percent co-loading of respondent-surrogate and pair-personal Q-sorts, percent concordance on healthcare-outcomes acceptability, or percent concordance on decision-control preference (*p* = 0.41 to 0.89).

Finally, there was no significant association between sex or religion on one hand and simulation or adjusted-simulation correlation score, mean surrogate’s decision-making confidence score, percent concordance on healthcare-outcomes acceptability, or percent concordance on decision-control preference on the other (*p* = 0.15 to 0.98). Age correlated significantly with surrogate’s decision-making confidence score (rho 0.24, *p* = 0.001, and healthcare-preferences’ familiarity score (rho − 0.21, *p* = 0.004) but not with simulation or adjusted-simulation correlation scores.

## Discussion

### Prediction accuracy of life-story narrative is inadequate

According to the substituted judgment standard, a surrogate decision-maker should make decisions in accordance with patient’s instructions, and if unknown, to predict the decisions that the patient would have made [2]. A national survey of US physicians found that they largely agree that surrogates should prioritize what the patient would have wanted over what they believe is in the patient’s best interests [28]. Nevertheless, a systematic review found low to moderate prediction accuracy that was improved with more extreme scenarios, under conditions of forced choice, and when family members were specifically directed to use substituted judgment [4]. Another systematic review found 68% prediction accuracy, no significant difference between patient-designated and legally assigned next-of-kin surrogates, and that prior discussion of patient’s treatment preferences did not significantly improve accuracy [5]. A recent study on African-American dialysis patients showed that 60% of surrogates were unsure how the patients would feel about continuing life sustaining treatment and only 35% patient-surrogate congruence in end-of-life goals of care, which had little association with surrogates’ decision-making confidence [6]. A more recent study found that 78% of surrogates (mainly white educated US women) focused more on patient’s well-being than on patient’s preferences [29].

Because of the demonstrated inadequate accuracy of substituted judgment and because of some philosophical challenges, other models of surrogate’s decision-making have been proposed [10, 12–20]. The patient’s life-story narrative model is based on understanding the personhood of the patient and focuses on respect for persons rather than narrowly on respect for autonomy, on authentic decisions rather than autonomous decisions, and on balancing rather than prioritizing [15–20]. Since the life-story narrative model simultaneously addresses multiple issues with a spectrum of answers rather than individual life-saving, binary-decision scenarios, previous results [4–7] showing inadequate accuracy of substituted judgment may not be applicable. The current study is the first to systematically evaluate surrogate’s accuracy in predicting life-story narrative.

Our respondents rank-ordered 47 opinion statements that covered a multitude of end-of-life issues, including, life quantity and quality, connectedness, transcendence, coping, information-disclosure, and decision-making. The statements were based on previous studies that explored general public, patients, and healthcare providers’ end-of-life priorities in various countries [30–38], including Saudi Arabia [9, 21, 22]. Although respondents expressed high degree of confidence in surrogate’s decision making and of familiarity with healthcare-preferences, they were still inadequate in predicting their participating family member’s life-story narrative. Only 41% of the respondent-surrogate Q-sorts co-loaded with pair-personal Q-sorts, whereas 56% co-loaded with respondent-personal Q-sorts. Further, correlation of individual statements’ ranking scores between respondent-surrogate and pair-personal Q-sorts was 0.42 compared to 0.62 between respondent-surrogate and respondent-personal Q-sorts. Consistent with previous results [39], the data indicate that surrogates are more likely to project their own life-story narrative than to simulate the life-story narrative of their family members.

In agreement with the results of previous studies conducted mainly in Europe and North America [4–8], prediction accuracy of decision-control preference and healthcare-outcomes acceptability were similarly inadequate, suggesting that the observation is not culture-related. Prediction accuracy was not associated with the degree of confidence in surrogate’s decision-making or of familiarity with healthcare-outcomes preferences. Although failure to find such an association may be due to narrow distribution of degree confidence and familiarity, true dissociation cannot be excluded since predictions of life-story narrative and healthcare-outcomes acceptability were best in the sibling-sibling subgroup, whereas confidence in surrogate’s decision-making was highest in the husband-wife subgroup.

### Reasons for inadequate prediction accuracy of life-story narrative

Inadequate prediction accuracy could be due to inadequate knowledge of the patient’s mind-set; to overwhelming emotions, such as not wanting to feel responsible for a loved ones’ bad outcome, a desire to pursue any chance of recovery, or choosing the “safer error” [7]; to internal struggle in prioritizing patient’s well-being/best interests, patient’s values/preferences, surrogate’s own values/preferences, family well-being/consensus, and social norms [14, 39, 40]; or to subconscious projection of surrogate’s interests, beliefs, and values. Since our study did not involve actual patients, overwhelming emotions would not be expected to have played a major role. Further, about 34% of respondent-surrogate Q-sorts did not co-load with either respondent-personal or pair-personal Q-sorts, suggesting that lack of family member-specific knowledge and/or internal struggle may be at least as important as subconscious projection.

Patients and their families may have the same priorities but may hold them at different hierarchies, which may be related to factors such as role-taking, sex, age, and social outlook. For example, ability to self-care was the most important and burden on family was the third most important end-of-life issues for patients, whereas amount of pain was the most important issue and burden on family was not an important issue for surrogates [41]. A study on advance care planning showed that men focused on minimally acceptable functional capacities while women took a broader view including meaningful times and places to die and psychological and economic burdens on families [42]. Another study showed that age, religiosity, and liberal-conservative social outlook explained 8, 5, and 5% of the variance in physicians attitude toward end-of-life decision-making [43]. We found that prediction accuracy varied according to the issue under consideration. For example, surrogates underestimated their family member’s preference for having a clergy present at the last moments and overestimated their preference for being informed about their impeding death by family/friends rather than the doctor, for not dying alone, and for discussing their illness with the family/friends present, suggesting that surrogates may overemphasize family-dependence and underemphasize transcendence. Of note, prediction accuracy of life-story narrative was not significantly associated with religion type. We also found notable differences among the three subgroups. For example, the importance of dying without having the body exposed was underestimated by surrogates in the parent-child and sibling-sibling subgroups but overestimated by surrogates in the husband-wife subgroup, whereas the importance of living longer regardless of medical condition was overestimated in the husband-wife and parent-child subgroups but not in the sibling-sibling subgroup.

Interestingly, prediction of life-story narrative, as measured by co-loading of respondent-surrogate and pair-personal Q-sorts, was best in the sibling-sibling subgroup, intermediate in the husband-wife subgroup, and lowest in the parent-child subgroup, suggesting an important role for age difference (and indirectly social outlook), in line with previous results [43]. Prediction accuracy was not significantly associated with age per se.

### Role of shared background in prediction accuracy of life-story narrative

For some issues, such as to die at peace with God and to maintain dignity there were very little intra-pair differences. Although this could be due to family member-specific knowledge, it is more likely related to shared background. In fact, although 41% of respondent-surrogate Q-sorts co-loaded with pair-personal Q-sorts, 56% co-loaded with respondent-personal Q-sorts. Further, when adjusted for correlation with respondent-personal ranking scores, correlation of statements ranking scores between respondent-surrogate and pair-personal Q-sorts dropped by 38% (from 0.42 to 0.26). This suggests that patient’ preferences can be, to a considerable extent, predicted without necessarily acquiring patient-specific knowledge, which give some credence to the population-based treatment indicator model and family-based model of surrogate’s decision-making. Interestingly, a preliminary US population-based treatment indicator was as accurate as surrogates in predicting patients’ choices [11]. Nevertheless, population-based indicators would not be expected to address patients as individuals or as members of a unique set of relationships.

It can be argued that family members are best suited to make surrogate decisions. People usually shares preferences, beliefs, and values more with their families than the general population. Further, family members usually have the best interests of the patient at heart, form a social unit that is essential in patient’s life, and have a desire to be involved in surrogate’s decision-making and to accept the associated moral responsibility [4]; and even though individual persons have diseases, whole families experience illnesses [44]. Furthermore, patients may care more about who makes the decision than the decision’s precise content, may not perceive deviations from their preferences as infarctions of their autonomy [45], and may belief that such decisions are not their right or (sole) responsibility, or are more important to their family than to them. In one study, 67% of US veterans preferred their advanced directives to be followed as general guidelines rather than binding instructions [46]. The extreme involvement of the family in surrogate’s medical decision-making is when the family makes decision collectively in a way that promotes the family’s interest; based on the principle of family autonomy or familism, a prevalent ethos in East Asia [12, 13]. Familism views the last journey of one’s life as a sharing process and holds that it is not ethically required to respect the patient’s prior wishes as self-determination is not relevant for adults who have lost the ability to make their own decisions. In the current study, only 7% of respondents preferred a “reliant”-decision-making (with or without considering their opinions), 43% preferred shared decision-making, and 50% preferred self-decision-making (with or without considering their family’s opinion). Further, prediction accuracy of decision-control preference was only 47%, consistent with the finding of a US study on seriously ill patients [25]. The results suggest that although family members may be advantaged in predicting the patient’s preferences and life-story narrative due to their shared background, familism-based surrogate’s decision-making was not preferred by most of our respondents. This is especially true for the sibling-sibling subgroup where 62% preferred self-decision-making. Of note, family-based surrogate’s decision-making can also be associated with substantial emotional burden during and after decision-making [40], in contrast to substituted judgment-based decision-making which may provide comfort by framing the decision as the patient’s own choice.

### Prediction accuracy of life-story narrative is more than explained by shared background

We found that adjusted prediction of life-story narrative (adjusted-simulation) is more than expected by chance or shared background. Under a 7-factor solution, about 14% of respondent-surrogate Q-sorts would be expected to co-load with pair-personal Q-sorts by chance, whereas in the current study 41% did. Further, adjusted-simulation correlation score was 0.26 (95% CI 0.24–0.28). Furthermore, adjusted-simulation correlation score was positively associated with expressed surrogate’s decision-making confidence. Consistent with our results, a study on surrogate utility estimation of healthcare and commodity items showed significant partial-correlations between what surrogates predicted for their partners and what the partners actually stated while controlling for their own utility judgment [47] and family surrogates were more accurate than physicians at predicting patients’ preferences [5].

Interestingly, in contrast to prediction of life-story narrative, predictions of decision-control preference and healthcare-outcomes acceptability were not associated with expressed surrogate’s decision-making confidence. Finally, co-loading of respondent-surrogate and pair-personal Q-sorts was higher in the sibling-sibling subgroup compared to the husband-wife and parent-child subgroups, suggesting that the common legally-determined next-of-kin hierarchy may not coincide with prediction accuracy of life-story narrative.

#### Study limitations

The following limitations should be taken into account when interpreting the results of this study. The study was based on a volunteer sample recruited from a single urban, academic center. Further, the sample had relatively high education level and lacked ethnic diversity. Therefore, the generalizability of our findings to other settings may be limited. The Q-set we used in this study was designed to capture life-story narrative in relation to end-of-life issues, thus our findings may not apply to other healthcare issues.

## Conclusions

The results of this study support the following main conclusions: 1) Intra-family predictions of life-story narrative, decision-control preference, and healthcare-outcomes acceptability were similarly inadequate and were not associated with expressed confidence in surrogate’s decision-making or familiarity with healthcare-outcomes preferences. 2) Even in non-stressful settings and despite being specifically directed, surrogates were more likely to project their own life-story narratives than to simulate those of their family members. 3) The accuracy of adjusted prediction of life-story narrative was more than expected by chance or by having similar background, positively associated with surrogate’s decision-making confidence, and highest in the sibling-sibling subgroup and lowest in the parent-child subgroup. 4) In contrast, prediction accuracy of decision-control preference and healthcare-outcomes acceptability were not associated with surrogate’s decision-making confidence or familiarity with healthcare-preferences. However, prediction accuracy of healthcare-outcomes acceptability was also highest in the sibling-sibling subgroup and worse in the parent-child subgroup.

The principle of individual autonomy and right of self-determination continue to dominate healthcare decision-making. The autonomy extension view proposes that people have the same authority over their future affairs as over their current affairs, which could be accomplished through advance directives or substituted judgment. The ethical validity of the autonomy extension view depends on philosophical understanding of autonomy, interests and their survival, and personhood [1, 3], and on surrogates’ ability to simulate patient’s preferences. Several studies [4–8] have documented inadequacy of substituted judgment in predicting individual choices. In the current study, we have shown that despite high self-reported surrogate’s decision-making confidence and healthcare-preferences familiarity, prediction of life-story narrative may be also inadequate. If our findings are supported by studies in other settings, one may conclude that more attention should be given to exploring other models of surrogate’s decision-making, such as the population-based treatment indicators model, familisim, and the Golden Rule, i.e., what surrogates would want for themselves [14], a fundamental concept of Judo-Christian and Islamic ethics.

## Additional files

Additional file 1: Q-Set Statements and Domains (DOC 34 kb)

Additional file 2: **Figure S1.** Husband-wife intra-pair differences in forced-ranking scores of 47 end-of-life opinion statements per predictor. Bars represent mean of 30 intra-pair differences (personal minus surrogate) in ranking scores (on a scale of 1 to 9). a, husbands predicting their wives preferences. b, wives predicting their husbands’ preferences. Full description of the statements is presented in Additional file 1. (PPTX 75 kb)

Additional file 3: **Figure S2.** Parent-child intra-pair differences in forced-ranking scores of 47 end-of-life opinion statements per predictor. Bars represent mean of 30 intra-pair differences (personal minus surrogate) in ranking scores (on a scale of 1 to 9). a, parents predicting their children preferences. b, children predicting their parents’ preferences. Full description of the statements is presented in Additional file 1. (PPTX 79 kb)

## Abbreviations

ANOVA: Analysis of variance
CI: Confidence interval
KFSH&RC: King Faisal Specialist Hospital and Research Center
REC: Research Ethics Committee
SD: Standard deviation
