# Supplementary material for: Prediction of life-story narrative for end-of-life surrogate’s decision-making is inadequate: a Q-methodology study
Source: BMC Med Ethics. 2019 May 3;20:28. doi: 10.1186/s12910-019-0368-8 (PMC6500001; doi:10.1186/s12910-019-0368-8)
Supplement: Supplementary file 2 — Figure S1. Husband-wife intra-pair differences in forced-ranking scores of 47 end-of-life opinion statements per predictor. Bars represent mean of 30 intra-pair differences (personal minus surrogate) in ranking scores (on a scale of 1 to 9). a, husbands predicting their wives preferences. b, wives predicting their husbands’ preferences. Full description of the statements is presented in Additional file 1. (PPTX 75 kb) [file 12910_2019_368_MOESM2_ESM.pptx]

## Slide 1
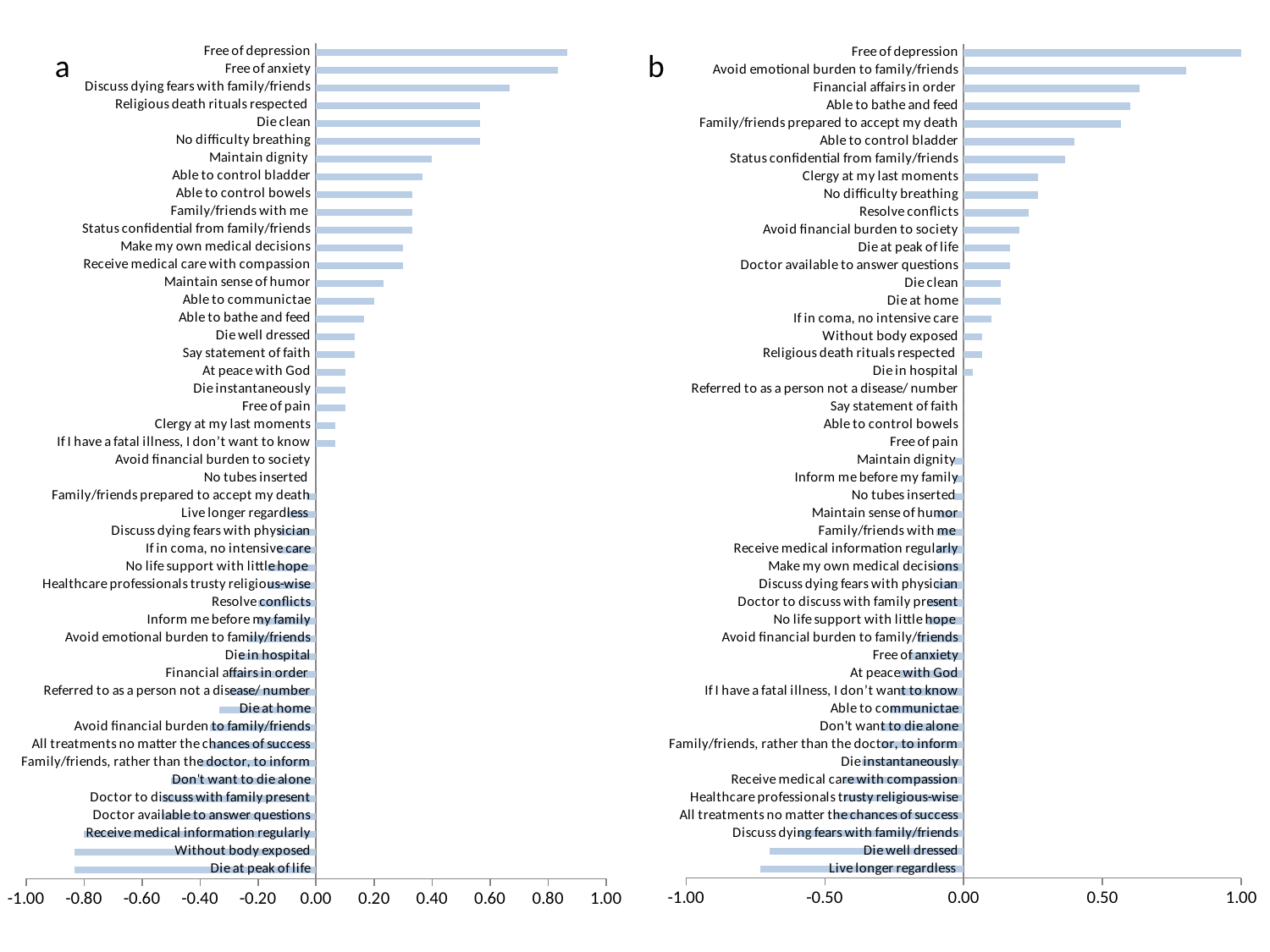

### Chart
| Category | Mean D |
|---|---|
| Die at peak of life | -0.8333333333333334 |
| Without body exposed | -0.8333333333333334 |
| Receive medical information regularly | -0.8 |
| Doctor available to answer questions | -0.5333333333333333 |
| Doctor to discuss with family present | -0.5333333333333333 |
| Don't want to die alone | -0.5 |
| Family/friends, rather than the doctor, to inform | -0.4 |
| All treatments no matter the chances of success | -0.36666666666666664 |
| Avoid financial burden to family/friends | -0.36666666666666664 |
| Die at home | -0.3333333333333333 |
| Referred to as a person not a disease/ number | -0.3 |
| Financial affairs in order | -0.3 |
| Die in hospital | -0.26666666666666666 |
| Avoid emotional burden to family/friends | -0.23333333333333334 |
| Inform me before my family | -0.2 |
| Resolve conflicts | -0.2 |
| Healthcare professionals trusty religious-wise | -0.16666666666666666 |
| No life support with little hope | -0.16666666666666666 |
| If in coma, no intensive care | -0.13333333333333333 |
| Discuss dying fears with physician | -0.13333333333333333 |
| Live longer regardless | -0.1 |
| Family/friends prepared to accept my death | -0.03333333333333333 |
| No tubes inserted | 0.0 |
| Avoid financial burden to society | 0.0 |
| If I have a fatal illness, I don’t want to know | 0.06666666666666667 |
| Clergy at my last moments | 0.06666666666666667 |
| Free of pain | 0.1 |
| Die instantaneously | 0.1 |
| At peace with God | 0.1 |
| Say statement of faith | 0.13333333333333333 |
| Die well dressed | 0.13333333333333333 |
| Able to bathe and feed | 0.16666666666666666 |
| Able to communictae | 0.2 |
| Maintain sense of humor | 0.23333333333333334 |
| Receive medical care with compassion | 0.3 |
| Make my own medical decisions | 0.3 |
| Status confidential from family/friends | 0.3333333333333333 |
| Family/friends with me | 0.3333333333333333 |
| Able to control bowels | 0.3333333333333333 |
| Able to control bladder | 0.36666666666666664 |
| Maintain dignity | 0.4 |
| No difficulty breathing | 0.5666666666666667 |
| Die clean | 0.5666666666666667 |
| Religious death rituals respected | 0.5666666666666667 |
| Discuss dying fears with family/friends | 0.6666666666666666 |
| Free of anxiety | 0.8333333333333334 |
| Free of depression | 0.8666666666666667 |
### Chart
| Category | Mean D |
|---|---|
| Live longer regardless | -0.7333333333333333 |
| Die well dressed | -0.7 |
| Discuss dying fears with family/friends | -0.6 |
| All treatments no matter the chances of success | -0.4666666666666667 |
| Healthcare professionals trusty religious-wise | -0.43333333333333335 |
| Receive medical care with compassion | -0.43333333333333335 |
| Die instantaneously | -0.36666666666666664 |
| Family/friends, rather than the doctor, to inform | -0.3 |
| Don't want to die alone | -0.3 |
| Able to communictae | -0.26666666666666666 |
| If I have a fatal illness, I don’t want to know | -0.23333333333333334 |
| At peace with God | -0.23333333333333334 |
| Free of anxiety | -0.2 |
| Avoid financial burden to family/friends | -0.16666666666666666 |
| No life support with little hope | -0.13333333333333333 |
| Doctor to discuss with family present | -0.13333333333333333 |
| Discuss dying fears with physician | -0.1 |
| Make my own medical decisions | -0.1 |
| Receive medical information regularly | -0.1 |
| Family/friends with me | -0.1 |
| Maintain sense of humor | -0.1 |
| No tubes inserted | -0.03333333333333333 |
| Inform me before my family | -0.03333333333333333 |
| Maintain dignity | -0.03333333333333333 |
| Free of pain | 0.0 |
| Able to control bowels | 0.0 |
| Say statement of faith | 0.0 |
| Referred to as a person not a disease/ number | 0.0 |
| Die in hospital | 0.03333333333333333 |
| Religious death rituals respected | 0.06666666666666667 |
| Without body exposed | 0.06666666666666667 |
| If in coma, no intensive care | 0.1 |
| Die at home | 0.13333333333333333 |
| Die clean | 0.13333333333333333 |
| Doctor available to answer questions | 0.16666666666666666 |
| Die at peak of life | 0.16666666666666666 |
| Avoid financial burden to society | 0.2 |
| Resolve conflicts | 0.23333333333333334 |
| No difficulty breathing | 0.26666666666666666 |
| Clergy at my last moments | 0.26666666666666666 |
| Status confidential from family/friends | 0.36666666666666664 |
| Able to control bladder | 0.4 |
| Family/friends prepared to accept my death | 0.5666666666666667 |
| Able to bathe and feed | 0.6 |
| Financial affairs in order | 0.6333333333333333 |
| Avoid emotional burden to family/friends | 0.8 |
| Free of depression | 1.1 |a
b
